# Zero-Dose Childhood Immunization in Conflict-Affected PSNP Districts of Ethiopia: A Comparative Cross-Sectional Study

**DOI:** 10.12688/gatesopenres.16374.1

**Published:** 2026-01-12

**Authors:** Fisseha Shiferie, Gashaw Andargie Biks, Kidist Negash, Dawit A Tsegaye, Gobena Seboka, Getnet Birhanu, Shibabaw Ewnetie, Tenaye Abate, Uche RalphOpara, Wondwossen A Alemayehu, Joseph Odu, Steven Neri, Frank DelPizzo, Kidist Belete

**Affiliations:** 1 Project HOPE Ethiopia Country Office, Addis Ababa, Ethiopia; 2 Project HOPE Headquarters, Washington, D.C., USA; 3 Project HOPE Africa Regional Office, Windhoek, Namibia; 4 Gates Foundation, Seattle, Washington, USA; 5 USAID Ethiopia Country Office, Addis Ababa, Ethiopia

**Keywords:** Maternal education, Vaccination dropout, Health equity, Conflict-affected settings, Zero-dose, Ethiopia, Immunization coverage, PSNP

## Abstract

**Background:**

Childhood immunisation is one of the most cost-effective public health interventions, preventing 4–5 million deaths annually. This study assessed the prevalence and determinants of zero-dose immunisation among children aged 12–35 months in conflict-affected districts implementing Ethiopia’s Productive Safety Net Programme (PSNP) to determine whether intervention and comparison areas are comparable before rollout of the enhanced service-integration model.

**Methods:**

A comparative cross-sectional survey was conducted among 4,099 mothers and caregivers of children aged 12-35 months in intervention and comparison PSNP districts. Data were collected using a structured questionnaire administered by trained enumerators. Multivariable logistic regression was used to identify factors associated with zero-dose status.

**Results:**

Zero-dose prevalence was 30% in intervention districts and 27% in comparison districts, with notable regional disparities: 22.5% in Amhara, 23% in Afar, and 39% in Tigray. Vaccination dropout showed a different pattern, with the highest rate in Afar (57.6%) and the lowest in Tigray (13.6%). DTP3 coverage was lowest in Afar (42.9%) and highest in Amhara (69.4%), while MCV1 coverage was highest in Tigray (83.8%), followed by Amhara (79.6%) and Afar (49.1%). In intervention districts, zero-dose status was significantly associated with region (AOR = 1.5; 95% CI: 1.1–2.2), lack of maternal education (AOR = 1.7; 95% CI: 1.1–2.7), unmarried status (AOR = 1.8; 95% CI: 1.0–3.2), older child age (24–35 months) (AOR = 3.7; 95% CI: 2.6–5.3), and longer distance to health facilities (AOR = 1.4; 95% CI: 1.0–2.2). In comparison districts, region, maternal education, and older child age remained significant predictors.

**Conclusions:**

The study highlights persistent inequities in immunisation coverage in conflict-affected settings. It also demonstrates comparability between intervention and comparison PSNP districts in zero-dose prevalence and its determinants. These baseline findings provide a foundation for attributing future post-intervention improvements to enhanced integration of health services within the PSNP framework.

## Background

Childhood immunisation is one of the most cost-effective public health interventions, preventing an estimated 4–5 million deaths globally each year by protecting against diseases such as measles, pertussis, tetanus, and diphtheria. Despite considerable progress in expanding coverage, stark inequities remain particularly in fragile, conflict-affected, and resource-constrained settings where access to routine immunisation services is often disrupted ( WHO, 2024a; Yibeltal et al., 2022; Sharma et al., 2021; Acharya et al., 2022; Lyons et al., 2024; Bantie et al., 2024).

In 2023, an estimated 14.5 million children were classified as zero-dose those who had not received the first dose of the diphtheria–tetanus–pertussis-containing vaccine (DTP1) by the end of their first year of life ( WHO, 2024b; Gavi, 2025). The prevalence of zero-dose children in underserved and conflict-affected areas of Ethiopia was 33.7%, compared to 44.0% in Somalia and 49% in Sudan. These children reflect not only gaps in immunisation programmes but also broader systemic exclusion from essential health services. The majority live in underserved and conflict-affected settings, where overlapping challenges including poverty, inadequate infrastructure, gender inequities, and humanitarian crises significantly hinder access to vaccination ( Biks et al., 2024a; Halane et al., 2025; Sabahelzain et al., 2025).

In Ethiopia, the Expanded Programme on Immunization (EPI) has improved vaccine availability and facilitated the introduction of new vaccines into the national schedule. However, substantial regional disparities in immunisation coverage persist. According to the 2019 Mini Demographic and Health Survey, the coverage of the third dose of DTP-containing vaccine (DTP3) was as low as 26% in Somali and 27% in Afar compared to 93% in Addis Ababa ( CSA & ICF, 2019). These disparities have been exacerbated by armed conflict, particularly in the Afar, Amhara, and Tigray regions, which has resulted in the destruction of health infrastructure, disruption of cold chain systems, displacement of communities, and loss of health workers. Consequently, routine immunisation services have been severely compromised, leaving many children unvaccinated ( Biks et al., 2024a; Shiferie et al., 2023; Biks et al., 2024b).

Launched in 2005, Ethiopia’s Productive Safety Net Programme (PSNP) is the country’s flagship social protection initiative, designed to address chronic food insecurity among vulnerable households through conditional or unconditional transfers of food or cash. In recent years, the programme has expanded to incorporate nutrition, health, and water, sanitation, and hygiene components, presenting opportunities to leverage PSNP platforms to improve health outcomes, including immunisation ( Abay et al., 2022; World Bank, 2020). Globally, there is increasing recognition of the potential for integrated service delivery and social protection systems to reach zero-dose children ( UNICEF, 2023).

Although the PSNP presents significant potential to improve equitable access to essential health services, there is a lack of empirical evidence on the effectiveness of deliberately integrating immunization services within its framework in conflict-affected settings. Understanding baseline zero-dose prevalence and its determinants across PSNP settings is essential for identifying equity gaps and informing strategies to reach underserved children.

In the current implementation approach, both intervention and comparison districts are PSNP districts; however, the intervention districts are planned to apply an enhanced model that integrates health components into the PSNP platform, while comparison districts continue routine PSNP activities. The enhanced model follows a “Hub and Spoke” approach in which selected health facilities serve as hubs to launch mobile and outreach teams, offer integrated reproductive, maternal, newborn, and child health (RMNCH) services on-site, and function as referral centers for clients from outreach points (spokes). The spokes represent community-based service delivery outlets located closer to target populations such as PSNP labor sites, cash/food distribution points, marketplaces, schools, and health posts.

This study examines and compares baseline prevalence and determinants of zero-dose immunization among children aged 12–35 months in intervention and comparison conflict-affected PSNP districts in Ethiopia. The objective of this baseline analysis is to determine whether the two groups are comparable before implementation, not to assess the impact of the enhanced model. Establishing this baseline comparability will be crucial for attributing post-intervention differences in future assessments to the enhanced integration of health services within the PSNP framework.

## Methods

### Study design

This study employed a comparative cross-sectional design and was conducted between 8 May and 30 July 2024.

### Study context

**Figure 1.**
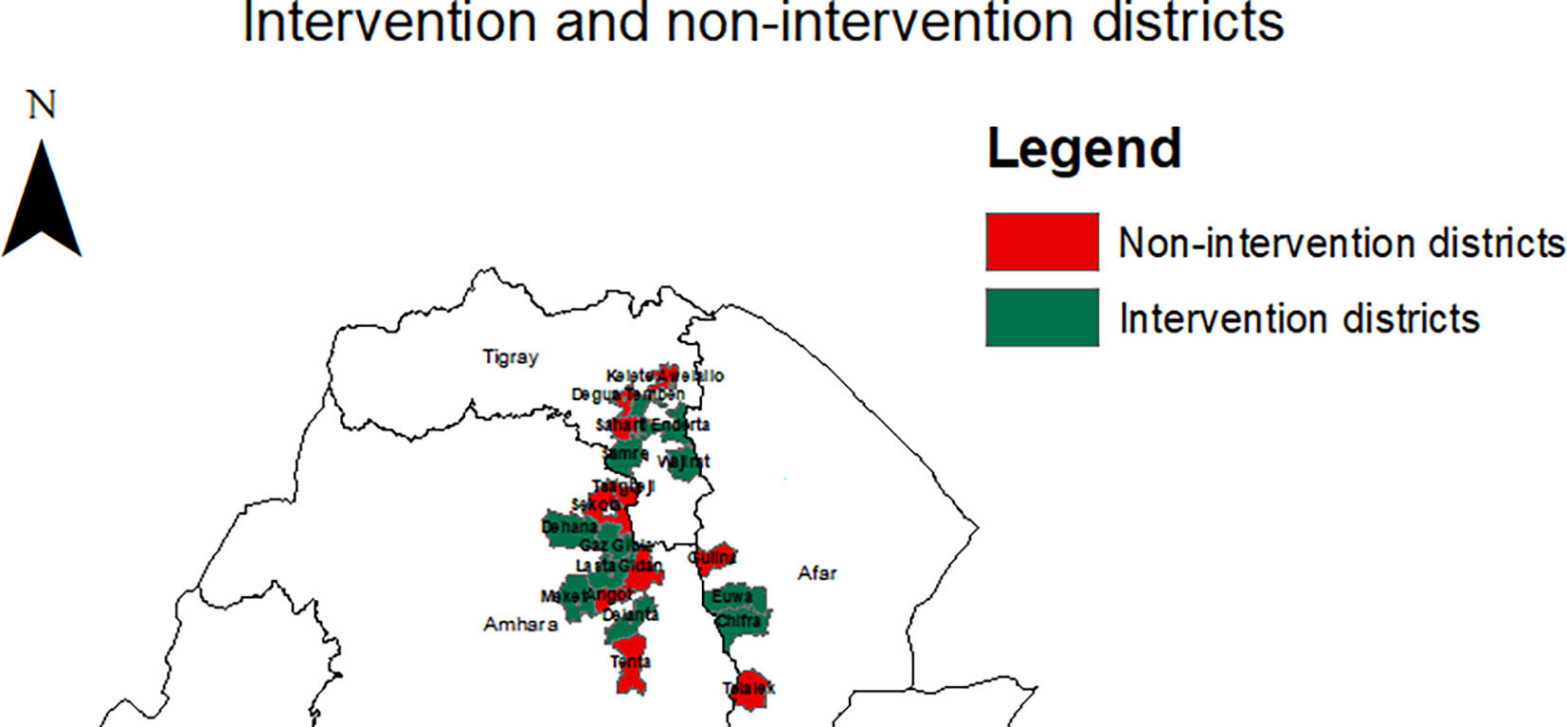
Map of intervention and comparison districts where the study was conducted.

The study was conducted in selected districts across the Amhara, Afar, and Tigray regions of Ethiopia. Twenty districts were included, comprising ten intervention districts (Chifra, Dehana, Delanta, Enderta, Ewa, Gaz Gibla, Lasta, Meket, Samre, and Wajirat) and ten comparison districts (Angot, Degua Temben, Gidan, Gulina, Kilte Awlalo, Seharti, Sekota Zuria, Telalek, Tsagibgi, and Tenta). Intervention districts were selected in consultation with regional and zonal health authorities based on the following criteria: relatively large population size, low utilisation of RMNCH services, absence of other implementing partners, and the presence of a comparatively strong health system to support integration with the PSNP ( Figure 1).

The selection of comparison districts was informed by a comprehensive analysis of socio-economic and health indicators, including access to basic services and relative levels of vulnerability within each region. This matching process aimed to ensure that comparison districts closely resembled intervention districts in key contextual characteristics. The findings of this study are expected to inform strategies to enhance the effectiveness of RMNCH outcomes and reduce disparities in service access and utilisation among the most vulnerable populations. By adopting a targeted, context-sensitive approach, the study contributes to the development of more effective and sustainable health interventions tailored to the specific needs of each community.

### Definition of terms

**Intervention districts**: Districts where enhanced integration of health services into the PSNP platform is being implemented through a “Hub-and-Spoke” model. In this approach, designated health facilities act as hubs offering integrated RMNCH services on-site, deploying mobile and outreach teams, and serving as referral centers for clients from community outreach points. The spokes are community-based service delivery outlets located closer to target populations including PSNP labor sites, cash/food distribution points, marketplaces, schools, and health posts where mobile and outreach services are provided.

**Comparison districts**: Districts where the PSNP is implemented without enhanced integration of health services into the PSNP platform.

### Source and study population

The three regions were selected based on their high concentration of PSNP beneficiaries and the substantial disruption to health systems caused by the recent conflict in northern Ethiopia. The study focused on rural households within selected PSNP implementation districts, specifically targeting those with at least one child under three years of age and/or a pregnant or lactating woman.

### Sample size

The sample size was calculated using Epi Info version 7, employing the two-population proportion formula. The calculation was based on key maternal and child health indicators, including: 59% prevalence of exclusive breastfeeding among children aged 0–5 months, 43% coverage of four or more antenatal care (ANC) visits, 50% skilled birth attendance, 36% coverage of women who gave birth in the past 35 months, 20% participation in growth monitoring for children aged 0–23 months, and 61% DTP3 vaccination coverage among children aged 12–35 months. The assumptions included a design effect of 2, statistical power of 80%, and a 10% non-response rate. Based on these parameters, the final required sample size was 4,066 households, with 2,033 PSNP beneficiary households allocated to each study arm (intervention and comparison).

The sampled households included children under 35 months of age, adolescents and youth, as well as pregnant and lactating women. However, during data collection, a total of 2,453 beneficiaries were interviewed in the intervention districts and 1,646 in the comparison districts. This deviation from the planned sample size was due to a limited number of eligible households identified in the comparison districts.

### Sampling procedure

A multi-stage cluster sampling technique was used to select study participants. The primary sampling units were districts in each study arm. From a sampling frame based on enumeration areas (EAs) delineated by the Central Statistical Agency of Ethiopia, 120 EAs (60 per arm) were randomly selected. The calculated sample size of 4,066 participants was proportionally distributed across EAs, assuming an average of 34 individuals per EA. All eligible children and other target participants within each selected EA were included without further sub-sampling to ensure comprehensive representation. The total sample size was sufficient to support subgroup analyses consistent with the study objectives and outcome indicators.

### Data collection

Data were collected using a structured questionnaire administered by trained data collectors and supervisors. The tool was developed based on a thorough review of validated instruments from similar studies to ensure contextual relevance and reliability. A total of 60 data collectors and 20 supervisors, all qualified public health professionals, were recruited and received five days of intensive training. The training covered the study objectives, data collection procedures, participant identification and assignment, questionnaire administration, and ethical considerations to ensure data quality and adherence to research ethics.

To ensure representative sampling, neighboring enumeration areas (EAs) were included when the required sample size could not be achieved within the initially selected clusters. In households with more than one eligible child, one child was randomly selected for inclusion to avoid intra-household clustering and ensure unbiased representation.

### Data quality

To ensure data quality, multiple measures were implemented. The questionnaire was translated into local languages and subsequently back-translated into English to verify consistency and linguistic accuracy. A pre-test involving 5% of the sample size was conducted in comparable settings to assess the clarity, relevance, and flow of the tool. All data collectors and supervisors underwent a five-day intensive training program. Additionally, data entry verification procedures were employed to minimize errors and enhance the overall accuracy and reliability of the data.

### Data management and analysis

Data completeness and consistency were reviewed at the end of each data collection day. Data collection was conducted electronically using a pre-tested, digitized questionnaire implemented on the CommCare platform ( Dimagi, Inc., 2022). The collected data were subsequently exported to STATA version 17.0 for statistical analysis. Frequency tables were generated to summarize the sociodemographic characteristics of respondents, and descriptive statistics were calculated for key variables, stratified by intervention and comparison districts.

To identify the factors associated with zero-dose status among children, both binary and multivariable logistic regression analyses were conducted separately for the intervention and comparison districts in conflict-affected regions of Ethiopia. The variables examined included region, marital status, maternal age, maternal education, age of the child, household income source, place of birth of the last child, number of children under five, distance to the nearest health facility, and paternal education.

All potential predictors with a p-value less than 0.02 in the binary logistic regression analysis were fitted into the final multivariable logistic regression model. Outputs were summarized using crude odds ratio and adjusted odds ratio (AOR) with 95% confidence interval. A p-value of ≤ 0.05 was considered statistically significant.

### Assessment of vaccination status

Children’s vaccination status was determined using a triangulation approach based on three data sources: caregiver reports, home-based records (i.e., vaccination cards), and facility-based records, in accordance with the World Health Organization’s *Practical Guide for Monitoring Immunization* ( WHO, 2015). In households where the mother or caregiver was able to present a vaccination card, immunization status was recorded based on direct card verification. In the absence of a card, the child’s vaccination status was assessed through maternal or caregiver recall. When a caregiver reported that the child had been vaccinated but could not provide the vaccination card, data collectors were instructed to verify the report by visiting the nearest health facility to review the child’s medical records. This multi-source validation aimed to enhance the accuracy of immunization status classification, particularly in settings where recordkeeping may be incomplete or disrupted due to conflict-related factors.

### Ethical considerations

The study was conducted in accordance with national and international ethical standards, including the Declaration of Helsinki. Ethical approval was obtained from the Institutional Review Board (IRB) of the Ethiopian Public Health Association (EPHA) (Approval Number: EPHA/OG/274/25). Administrative clearances were also secured from the respective Regional Health Bureaus (RHBs) to ensure compliance with local regulations.

Data collection commenced only after obtaining informed written consent from all participants, who were fully informed about the study’s purpose, procedures, and their right to withdraw at any time without penalty. For illiterate participants, informed consent was obtained through an oral explanation of the purpose of the study, procedures, potential risks, and benefits in the presence of an impartial witness. After confirming understanding and voluntary agreement to participate, the participant provided a thumbprint, and the witness signed the consent form on their behalf.

All personnel with access to the survey data completed internationally recognized training on the protection of human research participants. Additionally, data collectors received comprehensive training on ethical principles in human subjects research to uphold the highest standards of ethical conduct throughout the study.

## Results

### Sociodemographic characteristics

The study included 4,099 mothers and caregivers, with nearly 60% residing in intervention districts. The Amhara region accounted for 60% of the respondents. Children aged 12 to 23 months made up 33% of the sample, and approximately one-third of the respondents were between 25 and 29 years of age. At the time of the survey, 48% of respondents in the intervention districts and 46.3% in the comparison districts were illiterate ( Table 1).

**Table 1.** Sociodemographic characteristics of respondents in intervention and comparison districts (n = 4,099).

Types of districts	Frequency (n)	%
Intervention	2,453	59.8
Comparison	1,646	40.2
	**Intervention districts, n(%)**	**Comparison districts, n(%)**
**Region**		
Afar	372 (15.2)	181 (11.0%)
Amhara	1,412 (57.6)	1,045 (63.5)
Tigray	669 (27.3)	420 (25.5)
**Age of respondents (in years)**		
15-19	86 (3.5)	45 (2.7)
20-24	461 (18.8)	296 (18.0)
25-29	775 (31.6)	525 (31.9)
30-34	547 (22.3)	330 (20.1)
35-39	434 (17.7)	343 (20.8)
40-44	124 (5.1)	87 (5.3)
45-49	26 (1.1)	20 (1.2)
**Age of the youngest child of the respondents (in months)**		
0-6	515 (21.0)	360 (21.9)
7-11	393 (16.0)	255 (15.5)
12-23	839 (34.2)	493 (29.9)
24-35	706 (28.8)	538 (32.7)
**Age of adolescents in the households, n = 1,153**		
10-14 Yrs	468 (65.4)	283 (64.8)
15-19 Yrs	248 (34.6)	154 (35.2)
**Educational status of respondents**		
Can't read and write	1,175 (47.9)	762 (46.3)
Informal education	36 (1.4)	21 (1.3)
Primary education	636 (26.0)	390 (23.7)
Secondary education	575 (23.4)	461 (28.0)
Tertiary education	31 (1.3)	12 (0.7)

Housewives constituted the majority of respondents in both groups, representing 53% in the intervention districts and 57% in the comparison districts. Among husbands, 66% in the intervention districts and 70% in the comparison districts were engaged in farming. In the Afar region, 15% of respondents in the intervention districts and 9% in the comparison districts were pastoralists. In both district groups, the husband was typically the head of the household, and approximately 80% of respondents were married ( Table 2).

**Table 2.** Sociodemographic characteristics of respondents by district type (n = 4,099).

Variables	Intervention districts, n (%)	Comparison districts, n (%)
**Primary source of income of respondents**		
Housewife	1, 295 (52.8)	936 (56.9)
Daily laborer	216 (8.8)	95 (5.8)
Merchant	118 (4.8)	79 (4.8)
Farmer	554 (22.6)	433 (26.3)
Pastoralist	12 (0.5)	19 (1.1)
Employed	155 (6.3)	44 (2.7)
Others	103 (4.2)	40 (2.4)
**Primary source of income of husband**		
Farmer	1, 266 (66.1)	920 (70.0)
Daily laborer	178 (9.3)	134 (10.2)
Merchant	44 (2.3)	30 (2.3)
Employed	86 (4.5)	74 (5.6)
Pastoralist	280 (14.6)	123 (9.4)
Others	61 (3.1)	33 (2.5)
**Head of the household, n = 3, 229**		
Husband	1,854 (96.8)	1,287 (98.0)
Wife	51 (2.7)	27 (2.0)
Others	10 (0.5)	0 (0.0)
**Marital status of respondents**		
Single	213 (8.7)	104 (6.3)
Married	1,915 (78.0)	1,314 (79.8)
Divorced	243 (9.9)	169 (10.3)
Separated	34 (1.4)	26 (1.6)
Widowed	48 (2.0)	33 (2.0)

### Knowledge and attitudes toward childhood vaccination

Participants’ knowledge about the appropriate time to initiate childhood vaccination was assessed. Only 13% of respondents in the intervention districts and 14% in the comparison districts correctly identified that vaccination should begin at birth. In both regions, a relatively high proportion of caregivers, 70% in the intervention districts and 71% in the comparison districts, reported having received information about possible adverse events following immunization (AEFI) within the past six months. Additionally, 93% of mothers in both groups reported no fear, doubt, or suspicion regarding vaccinating their children. Consequently, 94% of respondents in the intervention districts and 91% in the comparison districts disagreed with the notion that some children should not be vaccinated ( Table 3).

**Table 3.** Knowledge, attitudes and practices related to the utilization of vaccination services in conflict-affected regions of Ethiopia, 2024.

Variables	Intervention districts, n (%)	Comparison districts, n (%)
**Knowledge about the time to start vaccination for children**		
At birth	329 (13.4)	230 (14.0)
In the first few weeks after delivery	1,133 (46.2)	863 (52.4)
In the first few months after delivery	872 (35.6)	497 (30.2)
Later	60 (2.4)	19 (1.1)
Do not know	59 (2.4)	37 (2.3)
**Message received about vaccination in the last 6 months**		
Yes	993 (40.5)	663 (40.3)
No	1,460 (59.5)	983 (59.7)
**Informed about possible adverse events of vaccination in the last 6 months**		
Yes	728 (29.7)	482 (29.3)
No	1,725 (70.3)	1,164 (70.7)
**Developing adverse events following immunization**		
Yes	1,304 (53.2)	762 (46.3)
No	1,149 (46.8)	884 (53.7)
**Types of adverse events developed**		
Injection site pain, redness and swelling	73 (5.6)	91 (12.0)
Injection site pain, redness & swelling AND Fever	616 (47.2)	294 (38.6)
Injection site pain, redness & swelling AND Body aches/rash	72 (5.5)	41 (5.4)
Fever	389 (29.8)	261 (34.3)
Body aches/rash	152 (11.7)	64 (8.4)
Others	2 (0.2)	11 (1.4)
**Belief that some children should not be vaccinated**		
Yes	40 (1.6)	24 (1.5)
No	2,299 (93.7)	1,504 (91.4)
Not sure	114 (4.7)	118 (7.2)
**Fear, doubt and suspicion about their child being vaccinated**		
Yes	124 (5.1)	78 (4.7)
No	2,270 (92.5)	1,527 (92.8)
Not sure	59 (2.4)	41 (2.5)
**Satisfaction about the vaccination services**		
Yes	2,162 (88.1)	1,441 (87.6)
No	291 (11.9)	205 (12.5)
**Household who had vaccination card or other documents**		
Yes	1,106 (71.6)	770 (74.7)
No	439 (28.4)	261 (25.3)
**Those who were able to show their cards/other documents**		
Yes	365 (33.0)	285 (37.0)
No	741 (67.0)	485 (63.0)
**Reasons for not showing their cards/documents**		
Kept in the health facility	115 (15.5)	30 (6.2)
Missed	588 (79.4)	440 (90.7)
Locked in another place	1 (0.1)	5 (1.0)
Others	37 (5.0)	10 (2.1)

### Availability of vaccination documentation

Among surveyed households, 72% in the intervention districts and 75% in the comparison districts reported possessing a vaccination card or related documentation. However, only 33% of respondents in the intervention districts and 37% in the comparison districts were able to physically present these documents at the time of the interview. A substantial proportion of caregivers reported having lost their child’s vaccination card or documents, with 79% in the intervention areas and 91% in the comparison districts unable to provide the records when requested. These findings underscore significant gaps in document retention and highlight the challenges of relying solely on home-based records for verifying immunization status, particularly in conflict-affected settings ( Table 3).

### Vaccination coverage, dropout rates, and zero-dose prevalence

In estimating vaccination coverage and zero-dose prevalence, maternal or caregiver recall was not considered due to concerns about recall bias, which can compromise data reliability. Instead, only documented sources including vaccination cards and verified facility records were used to classify vaccination status. For the purpose of this study, zero-dose children were defined as those who had not received DTP1 ( Gavi, 2025). Vaccination coverage was measured as the proportion of children who received DTP3, while vaccination dropout was defined as the proportion of children who received DTP1 but did not complete DTP3.

Based on these definitions, zero-dose prevalence was 30% in intervention districts and 27% in comparison districts. Across regions, prevalence ranged from 22.5% in Amhara to 23% in Afar and 39% in Tigray. Furthermore, zero-dose prevalence was consistently higher among children aged 24–35 months compared to those aged 12–23 months, suggesting a cumulative effect of service interruption over time. In contrast, vaccination dropout rates presented a different pattern. The Dropout rate was highest in Afar at 57.6%, whereas Tigray recorded a relatively low dropout rate (13.6%) ( Table 4, Table 5).

**Table 4.** Prevalence of zero-dose and vaccination coverage in conflict-affected regions of Ethiopia, 2024.

Variables	Intervention districts, n (%)	Comparison districts, n (%)
**Zero-dose children (DTP1 as indicated on immunization card or verified at health facility)**		
Zero-dose	198 (30)	110 (27)
Not zero-dose	1,086 (70)	762 (73)
**Zero-dose prevalence by age**		
12-23 months	81 (19.1)	47 (18.7)
24-35 months	117 (48.4)	63 (38.9)
**MCV1 coverage**		
Not vaccinated	107 (21.8)	67 (20.5)
Vaccinated	385 (78.3)	260 (79.5)
**MCV2 coverage**		
Not vaccinated	323 (66.0)	177 (54.3)
Vaccinated	166 (34.0)	149 (45.7)
**DTP3 coverage**		
Not vaccinated	371 (38.4)	263 (36.2)
Vaccinated	935 (61.6)	632 (63.8)
**HPV vaccination**		
Yes	452 (63.1)	291 (66.6)
No	264 (36.9)	146 (33.4)

**Table 5.** Regional comparison of zero-dose prevalence and immunization coverage, 2024.

Variables	Region		
	Afar (%)	Amhara (%)	Tigray (%)
Zero-dose prevalence (missing DTP1)	23.0	22.5	39.0
Zero-dose prevalence by age			
12-23 months	15.9	13.8	28.9
24-35 months	41.2	38.6	52.7
DTP1 coverage	77.0	77.5	61.0
MCV1 coverage	49.1	79.6	83.8
MCV2 coverage	7.2	40.8	41.4
DTP3 coverage	42.9	69.4	54.8
DTP dropout rate	57.6	22.9	13.6

Regarding overall DTP3 coverage, the study found rates of 42.9% in Afar, 69.4% in Amhara, and 54.8% in Tigray. Additionally, measles-containing vaccine first dose (MCV1) coverage was 78.8% overall, with regional rates of 49.1% (Afar), 79.6% (Amhara), and 83.8% (Tigray). Coverage of the measles-containing vaccine second dose (MCV2) was considerably lower, particularly in Afar, with only 7.2% coverage compared to 40.8% in Amhara and 41.4% in Tigray ( Table 5).

### Regional disparities in health service uptake

Analysis of selected indicators at the regional level revealed notable disparities in the uptake of healthcare services across the three study regions. The Tigray region demonstrated lower utilization of key health services compared to Afar and Amhara, including reduced coverage of DTP3. Tigray also recorded the highest prevalence of zero-dose children, defined as children who have not received DTP1, indicating significant gaps in the initial reach of routine immunization services.

While Afar exhibited relatively better initial vaccine uptake (as measured by DTP1 coverage), it had the highest dropout rate, the largest discrepancy between DTP1 and DTP3 coverage, suggesting substantial challenges in service continuity and follow-up in this region.

These regional differences underscore the importance of context-specific strategies to address both initial access to and continuity of health services, particularly in settings affected by conflict and systemic disruptions ( Table 5).

### Determinants of zero-dose status in conflict-affected regions

In the binary logistic regression, significant drivers of zero-dose status in the intervention group included region, marital status, maternal education, child’s age, and distance to the health facility. In the comparison group, region and age of the child emerged as significant factors.

The multivariable logistic regression analysis further confirmed these associations. In the intervention group, the odds of having a zero-dose child were significantly higher among respondents from certain regions (AOR = 1.5; 95% CI: 1.1–2.2), mothers with no formal education (AOR = 1.7; 95% CI: 1.1–2.7), those who were unmarried (AOR = 1.8; 95% CI: 1.0–3.2), mothers of older children (24–35 months) (AOR = 3.7; 95% CI: 2.6–5.3), and those who reported greater distance to a health facility (AOR = 1.4; 95% CI: 1.0–2.2) ( Table 6).

**Table 6.** Binary and multivariable logistic regression analysis for baseline assessment of zero-dose between intervention and comparison PSNP districts, 2024.

	Intervention group				Comparison group			
Variables	Zero-dose		Odds ratio		Zero-dose		Odds ratio	
	Yes, n (%)	No, n (%)	COR (95% CI)	AOR (95% CI)	Yes	No	COR (95% CI)	AOR (95%CI)
**Region**								
Afar	32 (6.8)	13 (6.6)	1.4 (0.7, 2.8)	1.1 (0.5, 2.3)	15 (4.9)	1 (0.9)	11.3 (1.5, 88.0) **	14.4 (1.8, 116.6) **
Amhara	283 (60.5)	97 (49.0)	1.7 (1.2, 2.4) **	1.5 (1.0, 2.2) **	203 (66.8)	44 (40.0)	3.5 (2.2, 5.5) **	3.8 (2.3, 6.3) **
Tigray	153 (32.7)	88 (44.4)	Ref.	Ref.	86 (28.3)	65 (59.1)	Ref.	Ref.
**Age of the respondents (in years)**								
15 – 24	89 (19.0)	35 (17.7)	1.3 (0.8, 2.1)	-	43 (14.1)	18 (16.4)	1.0 (0.5, 1.9)	-
25 – 34	274 (58.6)	111 (56.1)	1.2 (0.8, 1.8)	-	175 (57.6)	57 (52.8)	1.3 (0.8, 2.1)	-
35 and above	105 (22.4)	52 (26.3)	Ref.	Ref.	86 (28.3)	35 (31.8)	Ref.	Ref.
**Educational status of respondents**								
Can’t’ read and write	174 (37.2)	96 (48.5)	Ref.	Ref.	123 (40.5)	51 (46.4)	Ref.	Ref.
Primary education	156 (33.3)	45 (22.7)	1.9 (1.3, 2.9) **	1.7 (1.1, 2.7) **	77 (25.3)	31 (28.2)	1.0 (0.6, 1.8)	1.6 (0.9, 3.0)
Secondary and above	138 (29.5)	57 (28.8)	1.3 (0.9, 2.0)	1.2 (0.8, 1.9)	104 (34.2)	28 (25.4)	1.5 (0.9, 2.6)	2.1 (1.2, 3.8) **
**Marital status of respondents**								
Not ever married	40 (8.6)	28 (14.1)	Ref.	Ref.	17 (5.6)	11 (10.0)	Ref.	Ref.
Married/Living together	368 (78.6)	130 (65.7)	2.0 (1.2, 3.4) **	1.8 (1.0, 3.2) **	236 (77.6)	82 (74.6)	1.9 (0.8, 4.1)	1.5 (0.6, 3.7)
Divorced, Separated/widowed	60 (12.8)	40 (20.2)	1.1 (0.6, 2.0)	1.1 (0.6, 2.1)	51 (16.8)	17 (15.5)	1.9 (0.8, 5.0)	2.3 (0.8, 6.4)
**Primary source of income of respondents**								
Farmer and housewife	362 (77.4)	147 (74.2)	1.2 (0.8, 1.7)	-	253 (83.2)	82 (74.6)	1.7 (1.0, 2.9)	-
Employed, daily laborer & merchant	106 (22.7)	51 (25.8)	Ref.	Ref.	51 (16.8)	28 (25.4)	Ref.	Ref.
**Age of the child (in months)**								
12 – 23	343 (73.3)	81 (40.9)	4.0 (2.8, 5.6) **	3.7 (2.6, 5.3) **	205 (67.4)	47 (42.7)	2.8 (1.8, 4.3) **	2.9 (1.8, 4.7) **
24 – 35	125 (26.7)	117 (59.1)	Ref.	Ref.	99 (32.6)	63 (57.3)	Ref.	Ref.
**Distance to health facility**								
Greater than 30 mins	363 (77.6)	139 (70.2)	1.5 (1.0, 2.1) **	1.4 (1.0, 2.2) **	207 (68.1)	74 (67.3)	1.0 (0.7, 1.7)	1.2 (0.7, 2.0)
Less than or equal to 30 mins	105 (22.4)	59 (29.8)	Ref.	Ref.	97 (31.9)	36 (32.7)	Ref.	Ref.
**Number of under five children**								
One	304 (65.0)	134 (67.7)	0.9 (0.6, 1.3)	-	205 (67.4)	66 (60.0)	1.4 (0.9, 2.2)	-
Two or more	164 (35.0)	64 (32.3)	Ref.	Ref.	99 (32.6)	44 (40.0)	Ref.	Ref.
**Place of birth**								
Home	201 (43.0)	82 (41.4)	Ref.	Ref.	131 (43.1)	46 (41.8)	Ref.	Ref.
Health facility	267 (57.0)	116 (58.6)	0.9 (0.7, 1.3)	-	173 (56.9)	64 (58.2)	1.0 (0.6, 1.5)	-
**Educational status of husband**								
Can’t’ read and write	247 (52.8)	111 (56.1)	Ref.	Ref.	140 (46.1)	59 (53.6)	Ref.	Ref.
Primary education	135 (28.9)	46 (23.2)	1.3 (0.9, 2.0)	-	78 (25.7)	30 (27.3)	1.1 (0.7, 1.8)	-
Secondary and above	86 (18.4)	41 (20.7)	0.9 (0.6, 1.5)	-	86 (28.3)	21 (19.1)	1.7 (1.0, 3.0)	-

^**^ P < 0.05, - shows variables not included in the final multivariable model.

In the comparison group, region (AOR = 3.8; 95% CI: 2.3–6.3), maternal education (AOR = 2.1; 95% CI: 1.2–3.8), and age of the child (AOR = 2.9; 95% CI: 1.8–4.7) were significantly associated with zero-dose status ( Table 6). The variance inflation factor (VIF) values ranged from 1.5 to 2.3, suggesting the absence of significant multicollinearity among the variables.

## Discussion

This study aimed to assess the vaccination status, dropout rates, and the prevalence and determinants of zero-dose children in PSNP implementation districts of Ethiopia. The findings revealed substantial gaps in immunization coverage, with a high prevalence of zero-dose children. The disparities were especially pronounced in Tigray, where prolonged conflict has severely disrupted routine immunization services. These results highlight the profound challenges in sustaining equitable immunization coverage in fragile and humanitarian settings, where the destruction of health infrastructure, displacement of communities, and interruptions in cold chain logistics undermine vaccine delivery. The findings emphasize the urgent need for comprehensive, context-sensitive interventions that address both immediate access to vaccines and long-term service continuity.

### Vaccination status and coverage

The findings reveal that a substantial proportion of children in conflict-affected regions of Ethiopia remain unvaccinated or under-vaccinated, with 30% of children in the intervention districts and 27% in the comparison districts classified as zero-dose, having not received DTP1. The highest zero-dose prevalence was observed in Tigray (39%), a region heavily impacted by ongoing conflict, widespread displacement, and the destruction of essential health infrastructure. These results are consistent with evidence from other fragile and conflict-affected settings, where health system disruptions, population mobility, and the breakdown of routine health services significantly hinder vaccine delivery and uptake. Studies have demonstrated that conflict exacerbates pre-existing inequities in access to immunization, particularly among marginalized and hard-to-reach populations ( Sabahelzain et al., 2025; Biks et al., 2024a, 2024b). Furthermore, in such settings, children often miss out on vaccination opportunities due to insecurity, lack of transportation, and the diversion of resources toward emergency responses ( Shiferie et al., 2024a; Nigatu et al., 2024).

In contrast to the high prevalence of zero-dose children, vaccination dropout rates exhibited considerable regional variation. The Afar region recorded the highest dropout rate at 57.6%, indicating that although a significant number of children received DTP1, a majority failed to complete the full immunization schedule (i.e., did not receive DTP3). This pattern suggests that while initial access to immunization may be relatively functional, substantial challenges persist in ensuring continuity of care and follow-up, which are critical for achieving full protection against vaccine-preventable diseases ( Shiferie et al., 2023; Nigatu et al., 2024). Factors contributing to high dropout rates in Afar likely include nomadic lifestyles, weak health system infrastructure, limited outreach capacity, and insufficient tracking mechanisms for defaulters.

Conversely, the Tigray region, despite having the highest zero-dose prevalence, recorded a comparatively low dropout rate of 13.6%. This finding could be partially attributed to focused outreach efforts or emergency immunization campaigns conducted during windows of relative stability, which may have prioritized reaching children with initial vaccine doses. Alternatively, it may reflect selective access where only a subset of the population, those with better access to services, could initiate and complete vaccination. These contrasting patterns between zero-dose and dropout rates highlight the importance of distinguishing between access and retention in immunization programs and the need for context-specific strategies tailored to each region’s unique barriers and facilitators of vaccine uptake.

The overall coverage of DTP3 demonstrated substantial regional disparities, ranging from 42.9% in Afar to 69.4% in Amhara and 54.8% in Tigray. While MCV1 coverage was relatively higher particularly in Tigray (83.8%) and Amhara (79.6%), the uptake of MCV2 remained considerably low across all regions, with the most concerning figure observed in Afar, where MCV2 coverage was only 7.2%. The higher coverage observed for MCV1 relative to DTP3 could be explained by several factors, including dropouts across the DTP vaccination series, the impact of supplementary measles campaigns that reach previously unvaccinated children and variations in vaccine stock or the timing of vaccination visits. This steep drop between MCV1 and MCV2 coverage highlights gaps in follow-up and continuity of routine immunization services. Several factors may contribute to this, including logistical barriers, caregiver fatigue, and weak tracking systems. Moreover, the relatively recent introduction of MCV2 into Ethiopia’s EPI in 2019 has posed integration challenges, particularly in underserved and conflict-affected settings where health systems are already fragile. The administration of MCV2 at 15 months of age, a time when contact with health services may diminish, further exacerbates the risk of missed opportunities for immunization ( Minta et al., 2024; Shiferie et al., 2024b; MoH Ethiopia, 2024).

### Knowledge, attitudes, and practices

Knowledge about the appropriate timing of childhood vaccinations was notably low in the study population, with only 13% of respondents in the intervention districts and 14% in the comparison districts correctly identifying that vaccination should begin at birth. This finding aligns with evidence from other low- and middle-income countries, where limited awareness and misinformation about immunization schedules continue to impede vaccine uptake ( Danso et al., 2023; GebreEyesus et al., 2021). On a more positive note, a substantial proportion of caregivers, 70% in both groups, reported receiving information about potential AEFI, suggesting that community outreach efforts related to vaccine safety may be having an impact. Nevertheless, the persistent gap in knowledge about the recommended timing of childhood vaccinations highlights the need for targeted communication strategies and community education campaigns to improve timely vaccine initiation. Interestingly, these findings contrast with a study conducted in Southern Ethiopia, where 71.5% of caregivers adhered to the national immunization schedule and vaccinated their children accordingly ( Hardido et al., 2024), pointing to regional variations in awareness and adherence that must be considered in program planning.

Interestingly, 94% of respondents in the intervention districts and 91% in the comparison districts disagreed with the notion that some children should not be vaccinated. This suggests that vaccine hesitancy was not a major barrier within the study population, an encouraging finding for future immunization efforts. However, significant challenges were observed in the retention and presentation of vaccination documentation. A large proportion of caregivers were unable to produce vaccination cards at the time of the interview, which complicates efforts to verify immunization status accurately. These findings underscore the operational difficulties of monitoring vaccination coverage in conflict-affected and resource-limited settings, where health records are often lost or incomplete ( Tareke et al., 2024; Edossa et al., 2024).

### Determinants of zero-dose status

The multivariable analysis identified several key determinants of zero-dose status across both intervention and comparison districts. In the intervention areas, significant predictors included region, maternal education level, marital status, age of the child, and distance to the nearest health facility. In the comparison districts, region, maternal education, and child’s age remained the most influential factors. These findings align with previous studies emphasizing the critical role of maternal education and physical access to healthcare services in shaping childhood immunization outcomes ( Dires et al., 2025; Hassan et al., 2025; Sako et al., 2023; Galadima et al., 2021). Educated mothers are more likely to understand the importance of timely vaccination, navigate health systems, and adhere to immunization schedules, while geographic barriers continue to limit service utilization in underserved and remote regions.

The finding that children of mothers with no formal education were more likely to be zero-dose is consistent with a broad body of evidence highlighting maternal education as a key determinant of child health outcomes, particularly vaccination uptake ( Gebreyesus and Tesfay, 2024; Geweniger and Abbas, 2020). Educated mothers are more likely to understand immunization schedules, recognize the importance of vaccines, and navigate health services effectively. Furthermore, the higher odds of zero-dose status among children aged 24–35 months, especially in the intervention districts, may reflect the cumulative impact of prolonged disruptions to routine immunization services, potentially exacerbated by conflict, displacement, or health system instability. This underscores the importance of implementing catch-up strategies and targeted outreach to older cohorts of children who may have aged out of standard immunization schedules without receiving their vaccines due to these systemic challenges.

Additionally, the study found that residing farther from a health facility was significantly associated with higher odds of a child being zero-dose. This aligns with existing evidence indicating that physical distance to healthcare services remains a critical barrier to immunization, particularly in rural, pastoralist, and conflict-affected settings. Insecurity in conflict-affected areas restricts mobility, disrupts outreach services, and contributes to staff shortages and vaccine stockouts in remote facilities ( Albers et al., 2022; Freeman et al., 2023; Okwaraji et al., 2012; Bangura et al., 2020; Obanewa and Newell, 2020; Nour et al., 2020; Hogan and Gupta, 2023). Geographic inaccessibility often translates into limited contact with health workers, increased travel costs, and reduced health-seeking behavior, all of which contribute to missed vaccination opportunities. To address these barriers, health systems should prioritize strengthening outreach services, deploying mobile vaccination teams, and expanding the role of community health workers in immunization delivery. These strategies are particularly vital in reaching underserved populations and ensuring equitable access to essential childhood vaccines.

## Conclusion and recommendations

This study highlights the significant challenges to achieving equitable vaccination coverage in conflict-affected regions of Ethiopia. High zero-dose prevalence, vaccination dropouts, and regional disparities in coverage point to the need for comprehensive, context-sensitive interventions that address both immediate access to vaccines and long-term service continuity. It also establishes the comparability of zero-dose prevalence and its determinants between intervention and comparison conflict-affected PSNP districts. This foundation is essential for enabling future evaluations to attribute post-intervention differences specifically to the enhanced integration of health services within the PSNP framework.

Key recommendations include

• Integrate vaccination services within existing MNCH platforms to increase access for children who are often missed.
• Strengthen maternal education on vaccination schedules through community health workers, mother support groups and health facility counselling to increase timely uptake of routine immunization services.
• Expand and institutionalize outreach services to remote and hard-to-reach areas, ensuring availability of vaccines, logistics and trained personnel to provide consistent and reliable service delivery.
• Furthermore, rebuilding health infrastructure and ensuring the availability of vaccination documentation will be critical in sustaining immunization efforts in conflict-affected regions.
• The variation in vaccination coverage and dropout rates underscores the need for tailored strategies that consider the unique challenges faced by each region. For instance, in conflict-affected areas such as Tigray, rebuilding health systems and ensuring access to basic immunization services should be prioritized.
• In regions like Afar, strengthen defaulter tracking and community-based health worker outreach are critical.
• Rebuild cold-chain infrastructure and documentation systems in conflict-affected areas.
• Design region-specific strategies: reduce dropout in Afar, expand initial reach in Tigray, sustain coverage in Amhara.

The study also contributes valuable insights for designing future vaccination strategies and policy interventions, emphasizing the importance of addressing the unique barriers faced by vulnerable populations in conflict zones. These findings should inform future efforts to increase vaccination coverage and reduce the incidence of zero-dose children in similar contexts.

### Strength and limitations

This study has several notable strengths. First, it targeted a vulnerable and understudied population, generating critical data to inform targeted public health interventions. Second, the inclusion of both intervention and comparison districts enabled meaningful comparisons and facilitated a better understanding of the contextual factors influencing zero-dose prevalence. Third, the study addresses a global priority, identifying and reaching zero-dose children, thereby contributing to the goals of the global immunization agenda, including the Immunization Agenda 2030 (IA2030). Fourth, the use of multivariable logistic regression allowed for the identification of independent predictors of zero-dose status, offering deeper insights into the determinants beyond simple associations. Finally, the findings are directly relevant for policymakers, particularly in supporting Ethiopia’s efforts to expand immunization coverage and strengthen service delivery in humanitarian settings.

This study also has several limitations. First, its cross-sectional design limits the ability to infer causal relationships between the identified determinants and zero-dose status. Second, while the findings provide valuable insights for similar humanitarian or conflict-affected settings, they may not be generalizable to urban areas, more stable regions of Ethiopia, or countries with differing health system structures. The smaller sample size in comparison districts, resulting from a lower number of eligible households, may introduce selection bias and reduce comparability with the intervention group. This imbalance could influence the observed differences in vaccination outcomes if the sampled populations differ on unmeasured characteristics.

The study’s setting in conflict-affected areas presents potential limitations. Population displacement and instability may have influenced sample representativeness and the accuracy of vaccination coverage estimates. In addition, difficulties in verifying facility-based immunization records due to disrupted services, damaged or missing records, and restricted facility access may have introduced some measurement error or reporting bias.

## Ethics approval and consent to participate

The study was conducted in accordance with national and international ethical standards, including the Declaration of Helsinki. Ethical approval was obtained from the Institutional Review Board (IRB) of the Ethiopian Public Health Association (EPHA) (Approval Number: EPHA/OG/274/25). Administrative clearances were also secured from the respective Regional Health Bureaus (RHBs) to ensure compliance with local regulations.

Data collection commenced only after obtaining informed written consent from all participants, who were fully informed about the study’s purpose, procedures, and their right to withdraw at any time without penalty. All personnel with access to the survey data completed internationally recognized training on the protection of human research participants. Additionally, data collectors received comprehensive training on ethical principles in human subjects research to uphold the highest standards of ethical conduct throughout the study.

For participants under the age of 18, consent was obtained from a parent or legal guardian.

## Data Availability

The dataset supporting the findings of this study has been publicly uploaded to Figshare and is accessible via the following: DOI: https://doi.org/10.6084/m9.figshare.29313149.v1 ( Shiferie, 2025). Data are available under the terms of the Creative Commons Attribution 4.0 International license (CC-BY 4.0).
